# Antipruritic Effect of Qingpeng Ointment on the Localized Nonexudative Eczema

**DOI:** 10.1155/2019/4961691

**Published:** 2019-04-15

**Authors:** Yan Li, Wei Xu, Linfeng Li, Ruina Zhang

**Affiliations:** Department of Dermatology, Beijing Friendship Hospital, Capital Medical University, Beijing 100050, China

## Abstract

**Objective:**

To evaluate the effectiveness and safety of Qingpeng ointment on eczema-associated pruritus.

**Trial Design and Method:**

This single center randomized double-blinded placebo-controlled trial enrolled 60 patients with nonexudative eczema, who were randomized at 1:1 ratio to the Qingpeng ointment and placebo control groups (n=30 in each group). The investigational and control ointment were applied on lesions twice daily for two weeks. Visual analogue scale (VAS) and pruritus symptom scores were used to assess pruritus severity, frequency, and duration. Eczema lesions were evaluated by eczema area and severity index (EASI) and lesion morphology scores. Subjects were evaluated after the first treatment and at the end of the first and second week.

**Results:**

The average age and disease duration were 50.1±13.5 years and 30.9±16.0 weeks, respectively. Baseline EASI and VAS scores were similar between the two groups. VAS scores of the Qingpeng ointment group were significantly lower than those of the placebo control group at 10 minutes and 30 minutes after the first treatment and the first and second week follow-up (all* P* < 0.05). The scores of pruritus severity, frequency, and duration were significantly lower in the Qingpeng ointment group than in the control group at the end of week 1 and week 2 of the study (all* P* < 0.05). The scores of lesion morphology at the end of week 1 of the study were not significantly different between the two groups, but the Qingpeng ointment group showed significantly lower score than the placebo control group at the end of week 2 of the study. No adverse event was observed in the study.

**Conclusion:**

Qingpeng ointment can effectively alleviate pruritus and reduce skin lesions in patients with nonexudative eczema. The antipruritic effects occurred early and at a greater magnitude than the effects on lesion attenuation.

## 1. Introduction

Eczema, as a common skin disease, is characterized by a complex etiology and apparent pruritus. The clinical manifestation of eczema is diverse, and eczema lesions can develop in the whole body and are usually symmetric and exudative [1]. Pruritus is one of the most typical symptoms of the disease. To relieve the pruritus, patients often scratch the lesions intensively and thus cause the thickening of the lesions, which further aggravates the pruritus. The itch-scratch cycle prevents the lesions from healing, results in a long disease course, and causes distress to patients [2, 3]. Eczema-associated pruritus damages the quality of life seriously and thus should be treated promptly [3, 4].

The neurophysiological mechanism of pruritus remains unclear. Recent studies have confirmed the contributions of various factors to pruritus, such as factors that induce pruritus, specific receptors to the factors, afferent nerve fibers, and the specific regions of the central nervous system where scratch reflex occurs [5, 6]. Keratinocytes, which are the predominant cell type of the skin, play an important role in pruritus development. Under pathophysiological conditions, keratinocytes can secrete various substances to induce pruritus, such as cytokines, amines, neuropeptides, nerve growth factors, papaverine, and eicosanoids. These endogenous substances stimulate mast cells to release histamine or directly stimulate the sensory receptors in the C-fiber to induce pruritus [7, 8].

Keratinocytes express multiple receptors that are specifically associated with pruritus, such as histamine receptor, neuropeptide receptor, neurological factor receptor, cannabinoid receptor, protease activated receptor-2, and transient receptor potential vanilloid subtype 1 [9, 10]. Histamine receptors are related to skin barrier function; and damaged skin barrier function is associated with eczema-associated pruritus, atopic dermatitis, and dry skin [9, 10]. Because of the complex etiology and pathophysiological mechanism, treatment to pruritus is challenging and there is currently no effective therapy [11]. Glucocorticoids are commonly used to treat eczema-associated pruritus in clinical practice. Because glucocorticoids can reduce skin lesions and relieve pruritus by inhibiting skin inflammation, they could cure some patients with eczema. However, long-term use of the drugs may cause adverse reactions, such as infection and skin atrophy, and pruritus often recurs rapidly after the drugs are discontinued. Thus, standard therapies for eczema are still lacking, and effective therapies to treat eczema-associated pruritus are unmet needs [12, 13].

Multiple recent articles have shown that the traditional Tibetan herb medicine, Qingpeng ointment, can treat dermatitis and eczema effectively [14–16]. However, the effects of the ointment on eczema-associated pruritus have not been studied. Qingpeng ointment is a mixture of Tibet herbs, including* Oxytropis falcata Bunge*,* Rheum lhasaense*,* Aconitum pendulum Busch*,* Chebulae Fructus *(without the kernel),* Fructus Terminaliae Billericae*,* Phyllanthus emblica Linn, *Benzoin*, Tinospora sinensis (Lour.) Merr.*, and artificial musk. The main chemical components of* Oxytropis falcata Bunge* are flavonoids, alkaloids, and steroids. The roots, rhizomes, and the whole plant of* Oxytropis falcata Bunge* can be used as herb medicine. The herb has anti-inflammatory, analgesic, wound-healing, hemostatic, and anti-itching effects. The 3-acetate aconitine from* Aconitum pendulum Busch* can inhibit some early symptoms of inflammation, such as capillary hyperpermeability, exudation, edema, and leukocytosis. The main chemical component of* Chebulae Fructus*,* Fructus Terminaliae Billericae*, and* Phyllanthus emblica Linn* is tannin. Galloyl-containing tannin can block or reduce the release of inflammatory factors and thus attenuate inflammation. The main chemical components of* Chebulae Fructus* are tannin and polyphenols. The water extracts of* Chebulae Fructus* can terminate the free radical cascade and have strong antiallergic and antioxidant effects. Extracts from the leave of* Phyllanthus emblica Linn* can inhibit the bacteria,* Staphylococcus aureus*,* Escherichia coli*, and* Candida albicans* in vitro, and thus can block the allergic reactions caused by those bacteria [15, 16]. Polypeptide protein, the predominant component of musk, can inhibit some adverse changes during early inflammation, such as leukocyte migration, capillary hyperpermeability, inflammatory exudation, and edema; the polypeptide protein can also inhibit inflammation through immune regulation [15–17]. Studies using animal model have shown that Qingpeng ointment can significantly inhibit histamine-induced pruritus and attenuate the skin symptoms of eczema in guinea pigs [17]. The mechanism underlying the beneficiary effects may be related to the drug-mediated inhibition of inflammatory cells and restoration of the homeostasis of inflammatory and anti-inflammatory factors [17]. The current randomized, double-blind, placebo-controlled study aims to evaluate the effectiveness of Qingpeng ointment on the pruritus caused by localized nonexudative eczema.

## 2. Methods

### 2.1. Study Design

This was a single center, randomized, double-blind, placebo-controlled study. The study protocol was approved by the Institutional Review Board of Beijing Friendship Hospital (Approval No. 2017-P2-159-01). All participants signed the informed consent form. The trial has been registered at May 17th 2018. The trial was registered at http://www.chictr.org.cn/enIndex.aspx and the trial identification number is ChiCTR1800016190.

### 2.2. Patients

All patients, who were diagnosed with nonexudative eczema in the Department of Dermatology of Beijing Friendship Hospital from May 2018 to September 2018, were screened. Inclusion criteria were age was 18-75 years; clinical symptoms met the diagnostic criteria for nonexudative eczema [18]; the skin lesion area was 1%-15% of total surface area; pruritus was moderate or severe; the score of visual analogue scale (VAS) for pruritus severity was ≥ 4; patients provided the informed consent and participated in this clinical trial voluntarily. Exclusion criteria were patients had severe liver and kidney disease, blood system disease, autoimmune disease, chronic severe infection, diabetes, mental diseases, malignant tumor, and/or other serious diseases that might affect the efficacy assessment of the investigational drug; patients had been using topical corticosteroid within two weeks of the enrollment interview; patients had been using systemic corticosteroid or other immunosuppressive agents (such as triptolide) within four weeks of the enrollment interview; patients had eczema on the face and skin folds; patients were allergic to Qingpeng ointment; patients did not follow the study protocol.

### 2.3. Randomization and Blinding

Computer-generated random number was used to allocate participants into the test and control groups at 1:1 ratio. A study nurse, who was blinded to the group allocation, assigned the participants to the testing or placebo control group using the random numbers. Both participants and investigators were blinded to the group allocation.

### 2.4. Intervention

The investigational drug was Qingpeng ointment provided by Tibet Qizheng Tibetan Medicine Co., Ltd. (Batch number 170901). The placebo control was sham ointment, which is the nonmedicine component of Qingpeng ointment. The sham ointment was also provided by Tibet Qizheng Tibetan Medicine Co., Ltd. According to the fingertip unit [19], Qingpeng and sham ointment were applied on lesions and then the treated areas were gently massaged for 2 minutes to allow ointment absorption. The ointment was applied twice daily for two weeks.

### 2.5. Outcome Measures

The effectiveness of the drug on pruritus and skin lesions was evaluated. The patients were followed up at the end of the first and second weeks of the study. VAS score for pruritus and pruritus symptom scores were evaluated before the treatment and at the first and second week follow-up visits (Table 1). VAS scores for pruritus were also obtained at 0, 5, 10, and 30 minutes after the first drug administration. Lesion severity was scored as 0 for no lesion, 1 for mild lesion, 2 for moderate lesion, and 3 for severe lesion. Lesion morphology was defined as erythema (E), edema/papule (I), epidermal exfoliation (Ex), and lichenification (L). Lesion area score was defined as 0 for 0%, 1 for 1% - 9%, 2 for 10% - 29%, 3 for 30% - 49%, 4 for 50% - 69%, 5 for 70% - 89%, and 6 for 90% - 100%. Eczema area and severity index (EASI) = head/neck lesion area score ×total head/neck lesion severity score (E+I+Ex+L) × 0.1 + upper limb lesion area score × total upper limb lesion severity score (E+I+Ex+L) × 0.2 + trunk lesion area score × total trunk lesion severity score (E+I+Ex+L) × 0.3 + lower limb lesion area score × total lower limb lesion severity score (E+I+Ex+L) × 0.4 [20].

Efficacy index = (EASI before treatment - EASI after treatment)/EASI before treatment × 100%. Complete cure was defined as efficacy index ≥ 90%. Significant improvement was defined as 60% ≤ efficacy index < 89%. Moderate improvement was defined as 20% ≤ efficacy index < 59%. No improvement was defined as efficacy index < 20% [13]. Effective rate = (the number of patients with complete cure + the number of patient with significant improvement)/total number of patients ×100%.

### 2.6. Safety Assessment

Adverse reactions were recorded by inquiring the subject and observation during the follow-up visits.

### 2.7. Sample Size

The significant level *α* = 0.05. Power (1-*β*) was 80%. In our preliminary studies, we found the effective rate of two-week treatment was 76.7% and 39.5% in Qingpeng ointment group and control group, respectively. The ratio between the two groups was 1:1. The sample size was 30 in each group based on the normal approximation method.

### 2.8. Statistical Analysis

The statistical software SPSS 16.0 was used for data analysis. Continuous variables are presented as mean ± standard deviation (SD). Pairwise* t*-test and repeated measures analysis of variance were used for intragroup comparison of continuous variables. Categorical variables are presented as rates. Intragroup comparison of categorical variables was analyzed by *χ*2 test. Multivariate analysis of variance was used for intergroup comparison.* P* < 0.05 was considered statistically significant.

## 3. Results

### 3.1. Baseline Data

**Figure 1:**
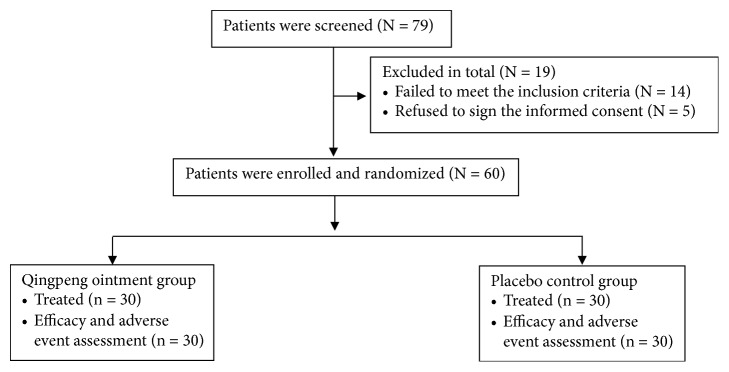
Patient flow diagram.

Participant flow diagram is presented in Figure 1. Sixty patients (32 men and 28 women) with localized nonexudative eczema participated in the study. The patients were randomized into the Qingpeng ointment group (n = 30) and placebo control group (n = 30) at 1:1 ratio. All the patients completed the trial. Patient baseline data are displayed in Table 2. The average age was 50.05±13.46 years, and the average disease duration was 30.93±16.00 weeks. The baseline data were not significantly different between the two groups.

### 3.2. VAS Score for Pruritus

The VAS scores before and after treatment are displayed in Table 3. For the Qingpeng ointment group, the VAS scores at 10 and 30 minutes after the first treatment were significantly lower than the score before the treatment (*P* < 0.001). The placebo control group also showed a significant reduction in the VAS score 10 minutes after the first treatment compared with the score before treatment, but did not decrease further at 30 minutes after the first treatment. Intergroup comparison demonstrated that VAS scores of the Qingpeng ointment group were significantly lower than those of the placebo control group at 10 minutes (4.03±1.43 vs. 4.97±1.52,* P* = 0.017) and 30 minutes (2.97±1.19 vs. 5.10±1.65, P < 0.0001) after the first treatment. In addition, VAS scores of the Qingpeng ointment group at the one-week and two-week follow-up visits were also significantly lower than those of the placebo control group (All* P* < 0.0001). These data suggest that Qingpeng ointment may attenuate eczema-pruritus effectively.

### 3.3. Pruritus Symptom Score and Lesion Morphology Score

**Figure 2:**
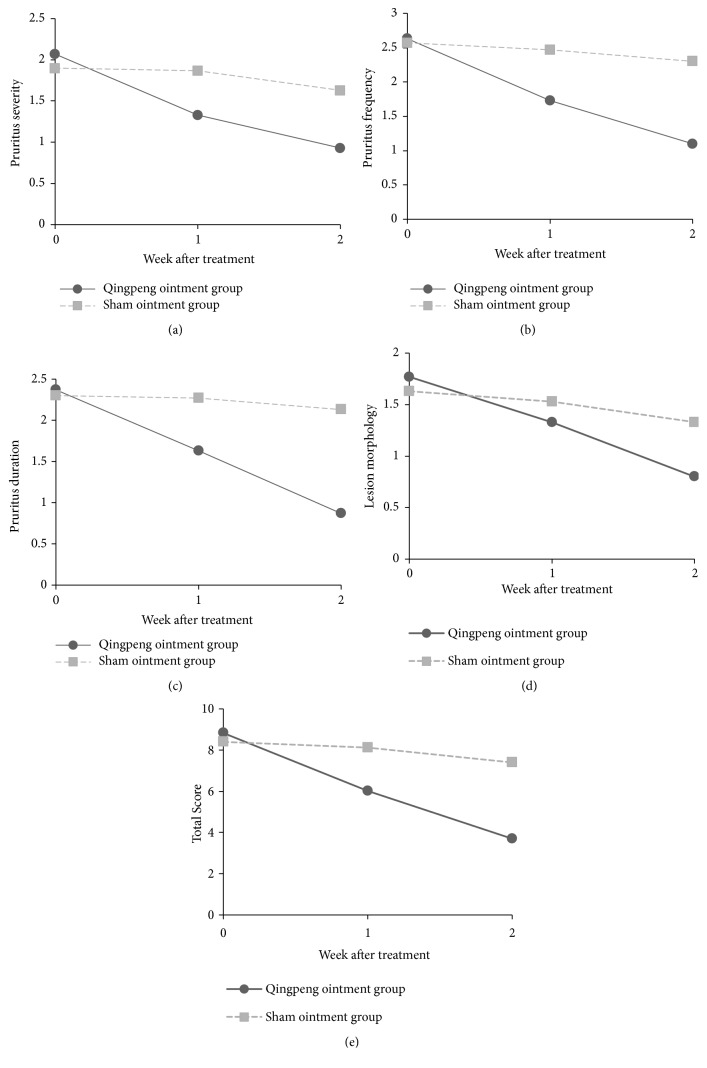
The scores of pruritus severity (a), frequency (b), duration (c), lesion morphology (d), and total score (e) changes.

The scores of pruritus symptoms and lesion morphology before and after treatment are presented in Table 4 and Figure 2. The scores of week 0 are baseline scores, which were not significantly different between the Qingpeng ointment and the placebo control groups. Notably, for the Qingpeng ointment group, the scores of pruritus severity, frequency, duration, lesion morphology, and total score decreased continuously and significantly from week 0 to week 2 (all* P* < 0.05). However, the placebo control group showed no such reductions. Intergroup comparison revealed that the total score and scores of pruritus severity, frequency, and duration were similar in the two groups before the treatment whereas were significantly lower in the Qingpeng ointment group than in the control group at the end of week 1 and week 2 of the study (all* P* < 0.05). The scores of lesion morphology before the treatment and at the end of week 1 of the study were not significantly different between the two groups, but the Qingpeng ointment group showed significantly lower score than the placebo control group at the end of week 2 of the study (*P* = 0.011). These results indicate that Qingpeng ointment-mediated attenuation in pruritus may occur earlier than the drug-mediated reduction in skin lesions.

### 3.4. Extent of Changes in EASI and VAS Scores

**Figure 3:**
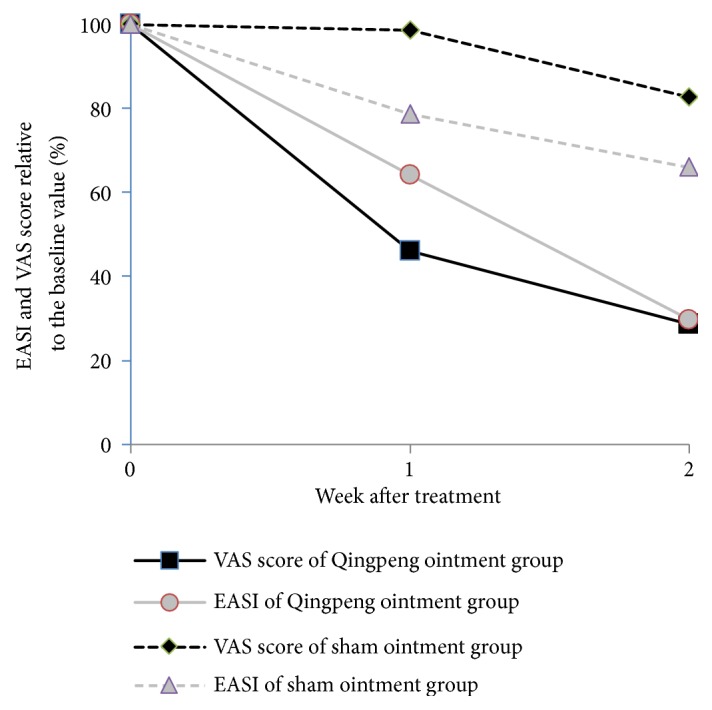
EASI and VAS score changes.

For the Qingpeng ointment group, the percentage of EASI at the end of week 1 and week 2 relative to the EASI before the treatment was 64.22% and 29.75%, respectively; the percentage of VAS score relative to the baseline score was 46.06% and 28.63%, respectively. For the placebo control group, the EASI% was 78.72% and 65.96%, respectively, and the VAS score% was 98.61% and 82.71%, respectively. The extent of reduction in EASI and VAS score in the Qingpeng ointment group was greater than that in the placebo control group (Figure 3). These results indicate that Qingpeng ointment-mediated attenuation in pruritus appears to show a greater magnitude than the drug-mediated reduction in skin lesions.

### 3.5. EASI and Effective Rate

The EASIs at week 0 and week 1 were similar in the two groups (Table 5). At the end of week 2 of the study, the Qingpeng ointment group had significantly lower EASI than the placebo control group (*P* < 0.0001). The effective rate of the Qingpeng ointment group was 43.33% and 76.67% at the end of week 1 and week 2 of the study, respectively (Table 5). The effective rates of the placebo control group were substantially lower than the Qingpeng ointment group although increased from 16.67% at the end of week 1 of the study to 36.67% at the end of week 2 of the study.

No adverse event was observed in the study.

## 4. Discussion

The current study found that Qingpeng ointment significantly reduced pruritus severity, frequency, duration, and lesion severity. Qingpeng ointment-mediated attenuation in pruritus occurred earlier than the reduction in lesions because the significant reduction in VAS score of pruritus occurred one week earlier than the significant reduction in EASI. These results suggest that Qingpeng ointment can inhibit eczema-associated pruritus without relying on the reduction in lesions. The effective rates of 43.33% at the end of week 1 and 76.67% at the end of week 2 of the study are consistent with the results of previous studies [14–16]. No adverse event was observed. Thus, the drug appears to be safe and effective for localized nonexudative eczema. Studies using animal model have shown that Qingpeng ointment can significantly inhibit histamine-induced pruritus and alleviate the eczema lesions in guinea pigs by inhibiting inflammation [21]. Our previous study has shown that Qingpeng ointment can effectively relieve eczema-associated pruritus in children [14]. Although the current clinical trial tested the effectiveness and safety of Qingpeng ointment on patients with moderate or severe eczema-associated pruritus, the drug should be also effective for patients with mild pruritus.

The molecular and cellular mechanism underlying the beneficial effects of Qingpeng ointment may be associated with the drug-mediated inhibition of the signal transduction pathway involved in pruritus development. Furthermore, vacuum homogenizing emulsification technology, super Micro-grinding technology, and unique oil-in-water cream form was used to manufacture Qingpeng ointment. Thus, the ointment can moisturize the skin. Compared with other traditional Tibetan medicine ointment, Qingpeng ointment can more effectively moisturize the skin and facilitate the drug absorption. The matrix of Qingpeng ointment consists of liquid paraffin, glycerin, water, and other moisturizing ingredients. Moisturizing the skin can improve skin barrier function by reducing KLK7 expression and thus effectively alleviate pruritus [15].

The current study showed that in the placebo control group, the VAS score of pruritus reduced significantly 5 minutes after the first administration of the sham ointment, suggesting the sham ointment also relieves pruritus at the first administration. The possible reason for this transient antipruritic effect from the sham ointment may be related to the cooling effects caused by evaporation of the water component of the ointment. This cooling effect might transiently mask the itching sensation. Thus, the VAS score of the placebo control group rose to the baseline levels at the end of the first week of the study. In contrast, VAS score of the Qingpeng ointment group reduced further at 10 and 30 minutes after the first treatment and remained at low levels during the study. The VAS score of Qingpeng ointment group reduced significantly at the end of the first week of the study, indicating a significant relieve of pruritus at the end of the first week, but lesion reduction occurred later. The lesion score reduced significantly at the end of the second week of the study and so did the EASI. Therefore, we believe that Qingpeng ointment may rapidly alleviate pruritus first and then reduce skin lesions by inhibiting inflammation and improving skin barrier function.

The current study showed that the magnitude of reduction in the VAS score was greater than that in the EASI, which suggests that the drug-mediated alleviation of pruritus had a greater magnitude than the effects of the drug on lesion reduction and that Qingpeng ointment can inhibit pruritus without depending on lesion reduction. Qingpeng ointment contains antipruritic ingredients from* Oxytropis falcata Bunge* and anti-inflammatory and antiallergic ingredients from* Chebulae Fructus*,* Fructus Terminaliae Billericae*, and musk.

The limitations of this study include a relatively small sample size and short follow-up time. Long-term follow-up is required to determine the long-term efficacy and the effects of Qingpeng ointment on eczema recurrence. The molecular and cellular mechanism underlying the benefit of Qingpeng ointment remains to be further investigated.

## Figures and Tables

**Table 1:** Pruritus symptom description and score.

Pruritus characteristics	Symptom description	Score
Severity	No itchy	0
	Occasionally itchy, no adverse effects on daily life	1
	Paroxysmal itchy with various intensity, adversely affecting sleep	2
	Intensive itchy, adversely affecting sleep and work seriously	3

Frequency	Not itchy	0
	Occasionally itchy, 1-2 episodes daily	1
	moderate itchy, 3-5 episodes daily	2
	Frequent itchy, > 5 episodes daily	3

Duration	Not itchy	0
	Last for less than 30 minutes	1
	Last for 30–60 minutes	2
	Last for more than one hour	3

Lesion morphology	No lesion	0
	Dry skin and desquamation	1
	Skin scratch and scar	2
	Skin hypertrophy and lichenification	3

Total Score= pruritus severity score + pruritus frequency score + pruritus duration score + lesion morphology score.

**Table 2:** Baseline data.

	Qingpeng Ointment Group N = 30	Sham Ointment Group N = 30
*Men/Women*	15/15	17/13

*Age (years)*		
Range, Min - Max	26 - 72	20 - 74
Mean ± SD	49.73 ± 13.82	50.36 ± 13.32

*Duration of Eczema (months)*		
Range, Min - Max	8-68	6-62
Mean ± SD	29.70±15.17	32.17±16.97

*EASI*		
Range, Min - Max	1.8 – 14.1	2.1 - 13.5
Mean ± SD	7.63 ±3.28	7.52±3.28

*VAS*		
Range, Min - Max	4-10	5-10
Mean ± SD	7.23 ±1.41	7.17±1.39

**Table 3:** VAS scores for pruritus before and after treatment.

	Qingpeng Ointment	Sham Ointment	P value
Before treatment	7.23 ±1.41	7.17±1.39	0.854
0 min after the first treatment	5.07±1.82	5.47±1.55	0.363
5 min after the first treatment	4.97±1.79	5.03±1.54	0.878
10 min after the first treatment	4.03±1.43	4.97±1.52	0.017
30 min after the first treatment	2.97±1.19	5.10±1.65	<0.0001
One-week follow-up visit	3.33±1.02	7.07±1.48	<0.0001
Two-week follow-up visit	2.07±1.05	5.93±1.11	<0.0001

Repeated measures analysis of variance was used to compare VAS scores at different time points. Multivariate analysis of variance was used to compare the two groups.

**Table 4:** Pruritus symptom and lesion morphology scores before and after treatment.

	Qingpeng Ointment	Sham Ointment	P value
*Pruritus Severity*			
Week 0	2.07±0.87	1.90±0.80	0.443
Week 1	1.33±0.48	1.87±0.78	0.002
Week 2	0.93±0.45	1.63±0.61	< 0.001
*Pruritus Frequency*			
Week 0	2.63±0.61	2.57±0.68	0.692
Week 1	1.73±0.74	2.47±0.68	< 0.001
Week 2	1.10±0.66	2.30±0.70	< 0.001
*Pruritus Duration*			
Week 0	2.37±0.76	2.30±0.75	0.734
Week 1	1.63±0.56	2.27±0.74	< 0.001
Week 2	0.87±0.35	2.13±0.68	< 0.001
*Lesion morphology Score*			
Week 0	1.77±0.73	1.63±0.85	0.517
Week 1	1.33±0.71	1.53±0.86	0.330
Week 2	0.80±0.55	1.33±0.96	0.011
*Total Score*			
Week 0	8.83±2.31	8.40±2.61	0.498
Week 1	6.03±1.45	8.13±2.43	< 0.001
Week 2	3.70±1.68	7.40±2.21	< 0.001

Repeated measures analysis of variance was used to compare the scores of pruritus severity, frequency, duration, lesion morphology, and total score at different time points. Multivariate analysis of variance was used to compare the two groups.

**Table 5:** EASI and effective rate.

	Qingpeng Ointment	Sham Ointment	P value
*EASI*			
Week 0	7.63 ±3.28	7.52±3.28	0.894
Week 1	4.90±3.04	5.92±3.20	0.209
Week 2	2.27±1.81	4.96±3.39	<0.0001

*Efficacy (No. of patients)*			
Week 1			
Complete cure	3	0	
Significant improvement	10	5	
Moderate improvement	5	12	
No improvement	12	13	
Effective rate (%)	43.33 (13/30)	16.67 (5/30)	

Week 2			
Complete cure	8	3	
Significant improvement	15	8	
Moderate improvement	4	9	
No improvement	3	10	
Effective rate (%)	76.67 (23/30)	36.67 (11/30)	

Repeated measures analysis of variance was used to compare EASI at different time points. Multivariate analysis of variance was used to compare the two groups.

## Data Availability

The datasets generated during and/or analyzed during the current study are available from the corresponding author upon reasonable request.
